# Clinical observation on the effect of Wuzhi soft capsule on FK506 concentration in membranous nephropathy patients

**DOI:** 10.1097/MD.0000000000018150

**Published:** 2019-11-27

**Authors:** Zhu Zhang, Xiaobei Lu, Leipeng Dong, Jiwei Ma, Xiaoguang Fan

**Affiliations:** a Department of Nephrology, Fuwai Central China Cardiovascular Hospital; b Department of Nephrology, People's Hospital of Zhengzhou, Zhengzhou; c Department of Nephrology, The people's Hospital of Xuchang, Xuchang; d Department of Nephrology, First affiliated Hospital of Henan university of traditional Chinese medicine, Zhengzhou, China.

**Keywords:** CYP3A5 gene polymorphism, membranous nephropathy, tacrolimus, Wuzhi soft capsule

## Abstract

The current research aimed to investigate the correlation between the effect of Wuzhi soft capsule (WZC) on FK506 concentration and CYP3A5 gene polymorphism in patients with membranous nephropathy (MN).

Seventy-five patients with idiopathic MN were enrolled and divided according to the expression of CYP3A5 gene metabolic enzyme into group A (CP3A5 metabolic enzyme function expression types CYP3A5∗1/∗1 type and CYP3A5∗1/∗3 type), and group B (non-expression type CYP3A5∗3/∗3 type). All patients were given oral administration of tacrolimus capsule at the initial dose of 1 mg for twice a day 1 hour before breakfast and dinner. Afterwards, the oral administration of WZC was added at the dose of 0.5 g for 3 times a day within half an hour after 3 meals.

The blood concentrations of FK506 in groups A and B were significantly higher than those before administration. Compared with that before administration, the FK506 blood concentration was increased by 3.051 ± 0.774 ng/ml after adding the WZC. Besides, the blood concentrations of FK506 in group A were lower than those in group B before and after administration; meanwhile, the 24 hours total urine protein and the biochemical indexes in both groups displayed no statistically significant difference. Only 1 case of diarrhea was observed, which was relieved after the reduction of tacrolimus.

Wuzhi soft capsule can significantly increase the blood concentration of FK506 in MN patients. Moreover, the CYP3A5 genotyping should be considered when WZC is used to increase the blood concentration of FK506.

## 1 Introduction

Tacrolimus (FK506), which belongs to the Calcineurin inhibitor (CNI), is a kind of macrolide antibiotic extracted from the fermentation liquid of Steptomyces tsukubaensis. It is the representative drug in the second generation immunosuppressor. FK506 can prevent and treat immune reaction, which has been increasingly used to treat some autoimmune diseases in recent years, such as rheumatoid arthritis, atopic dermatitis and membranous nephropathy (MN).^[1]^ However, FK506 is associated with the disadvantages of narrow therapeutic index, obvious individual differences in pharmacokinetics and toxicology, and high price, which have brought certain economic burdens on the long-term clinical application of patients. In the body, FK506 is mainly absorbed, excreted and re-absorbed through the translocator P-glycoprotein (P-gp) in the liver and small intestine; moreover, it is metabolized and decomposed through the cytochrome P450.^[2]^ Thus, it can be figured out that, the cytochrome P4503A (CYP3A) and P-gp gene polymorphism are the important factors affecting the blood concentration of FK506.^[3]^

Wuzhi soft capsule (WZC) is the mixture of the active ingredients (deoxyschizandrin) prepared from the kadsura longepedunculata through the alcohol extraction method, including schizandrin, schisandrol and schisandrin.^[4]^ It is verified in study through constructing the Caco-2 cell model that, deoxyschizandrin, schisandrol and schisandrin can suppress the P-gp mediated FK506 excretion, among which, deoxyschizandrin has the most potent effect on suppressing P-gp.^[5]^ Some scholars confirm in their study that,^[6]^ WZC can inhibit the metabolism of P-gp and CYP3A to enhance the FK506 concentration. At present, some research reports that CYP3A5 gene polymorphism will affect the blood concentration of FK506 in patients undergoing renal transplantation.^[7–9]^ However, no in-depth study is reported on the role of MN patients and CYP3A5 gene polymorphism in the influence of WZC on enhancing the FK506 blood concentration. Thus, this study aimed to observe the correlation of early WZC administration on enhancing the FK506 blood concentration and the CYP3A5 gene polymorphism in MN patients, so as to provide foundation for individualized treatment in clinic.

## 2 Methods

### 2.1 Objects of study

Patients visiting at the Nephrology Department of the First Affiliated Hospital of Henan University of Traditional Chinese Medicine from January 2017 to January 2018, manifesting as nephrotic syndrome (NS) and pathologically confirmed as MN through renal biopsy were selected. A total of 75 patients were enrolled in this study, including 37 males and 38 females, with the age of 18 to 75 years and the body weight of 43 to 80 kg. The inclusion criteria were as follows:

(1) patients aged between 18 and 75 years, with Han nationality, regardless of male or female;
(2) patients confirmed as MN through renal biopsy pathological examination;
(3) Scr<140.0 μmol/L;
(4) patients that had not taken any agent that might affect CYP3A enzyme (including diltiazem, ketoconazole, berberine and biphenyl diester) and P-gp at least within 1 month prior to the administration of FK506; and
(5) those who were willing to participate in the experiment and had signed the informed consent.

The exclusion criteria were as follows:

(1) patients with secondary MN, diabetes and diabetic nephropathy (DN);
(2) patients with severe infection, liver and kidney impairment (ALT or AST is more than 5 times the upper limit of the normal range, renal creatinine clearance <20 m L/min, respectively), gastrointestinal bleeding as well as other complications, and were immunosuppressor intolerant;
(3) pregnant women, breast-feeding women and females with fertility demand; and
(4) those allergic to drugs.

This study conformed to the ethics standard from the Ethics Committee of the First Affiliated Hospital of Henan University of Traditional Chinese Medicine, and all patients were informed and had signed the informed consent to participate in the experiment.

### 2.2 Treatment methods

**Figure 1:**
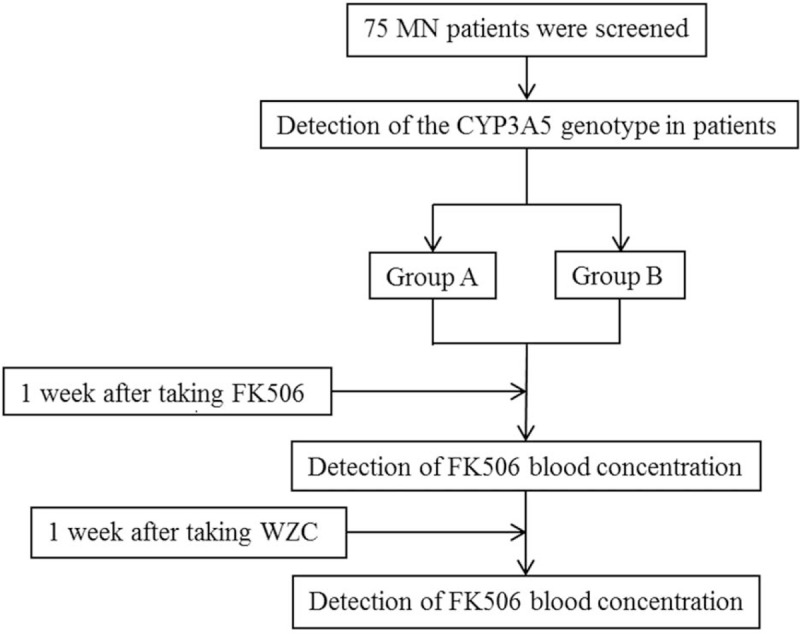
A flow chart of the experimental method. Note: Group A: CP3A5 metabolic enzyme functional expression type CYP3A5∗1/∗1 type and CYP3A5∗1/∗3 type. Group B: CP3A5 metabolic enzyme non-expression type CYP3A5∗3/∗3 type.

Participants in this study first underwent genetic testing and were then divided into 2 groups according to the CYP3A5 metabolic enzyme genotypes, which resulted in the different metabolic rates of FK506. FK506 was metabolized faster in CYP3A5∗1/∗1 and CYP3A5∗1/∗3, and the FK506 concentration could not be maintained in these 2 CYP3A5 genotypes. Whereas the FK506 concentration could be maintained in CYP3A5∗3/∗3 type due to the low metabolic rate. Therefore, group A included CYP3A5∗1/∗1 type and CYP3A5∗1/∗3 type, and group B included CYP3A5∗3/∗3 type. Afterwards, the patients were given oral administration of tacrolimus capsule at the initial dose of 1 mg for twice a day 1 hour before breakfast and dinner (at an interval of 12 hours) on an empty stomach. 1 week later, the oral administration of WZC was added at the dose of 0.5 g for 3 times a day within half an hour after 3 meals. FK506 (Astellas Pharma Co. Limited, Ireland, Registration number: J20150101, specification: 0.5 mg) was given orally at the initial dose was 1 mg for twice a day (generally, the initial dose of tacrolimus was 0.05 mg/kg/d) on an empty stomach 1 hour before breakfast and dinner, respectively (at an interval of 12 hour). The tacrolimus dose was adjusted according to the FK506 blood concentration, so that the latter was maintained within the treatment window of 4 to 8 ng/mL. WZC (Sichuan Hezheng Pharmaceutical, approval number: Z10983013, specification: each capsule contained 11.25 mg of deoxyschizandrin) was given orally at the initial dose of 0.5 g within half an hour after 3 meals (generally, the normal initial dose of WZC was 3 g/d), and the dose was not adjusted during the treatment. Corbrin capsule was also given orally at the dose of 1.5 g 30 minutes after three meals. No other immunosuppressor or cytotoxic drug was allowed during the treatment (Fig. 1).

### 2.3 FK506 concentration detection and gene polymorphism detection

The 2 ml fasting venous blood was collected from patients before taking WZC, as well as in the morning on the following day after 1 week of WZC treatment (12 hours after the last administration), and the blood samples were sent to the Kidney Disease Diagnostic and Treatment Center Laboratory to detect the tacrolimus blood concentration. The data were derived from the Kidney Disease Diagnostic and Treatment Center Laboratory of the First Affiliated Hospital of Henan University of Traditional Chinese Medicine.

### 2.4 Statistical method

The SPSS22.0 analysis software was adopted for statistical processing. The quantitative data were expressed as mean ± standard deviation (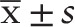), intergroup data conforming to normal distribution were analyzed using independent sample *t* test, while those not conforming to normal distribution were analyzed by rank sum test. Intragroup data were compared through paired *t* test. A difference of *P* < .05 was deemed as statistically significant.

## 3 Results

### 3.1 General clinical data

A total of 75 patients were enrolled in this study, including 6 of CYP3A5∗1/∗1 type, 37 of CYP3A5∗1/∗3 type, and 32 of CYP3A5∗3/∗3 type. In addition, there were 43 cases in group A (CYP3A5∗1/∗1 type and CYP3A5∗1/∗3 type), and 32 in group B (CYP3A5∗3/∗3 type), including 37 males and 38 females. The age of patients ranged from 18 to 75 years, and the average age in group A was (49.42 ± 14.29) years, while that in group B was (45.22 ± 12.82) years. The body weight of patients ranged from 43 to 80 kg, and the average body weight in group A was (49.42 ± 14.29) years, while that in group B was (45.22 ± 12.82) kg. Among the 75 patients, 22 cases were at the age group of 40 to 49 years, which had accounted for the highest proportion (29.33%).32.56% patients in group A were at the age group of 40 to 49 years, while that in group B was 25.00%. With regard to staging, 15 cases in group A were at stage I, 24 at stage II, 4 at stage III, and 0 at stage IV; meanwhile, 10 in group A were at stage I, 21 at stage II, 1 at stage III, and 0 at stage IV. Patients at pathological stage II had taken up the highest proportion (60.00%). Differences in the general data (sex, age and body weight), as well as general clinical indexes, such as 24 hours urine protein quantification, ALB, Scr, AST, ALT and GLU, between two groups were not statistically significant (*P* > .05) (Tables 1 and 2).

**Table 1:**
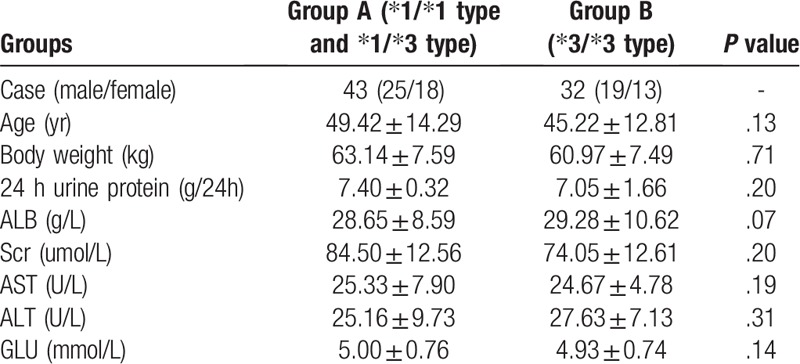
General condition and baseline levels of all indexes of patients in both groups (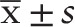).

**Table 2:**
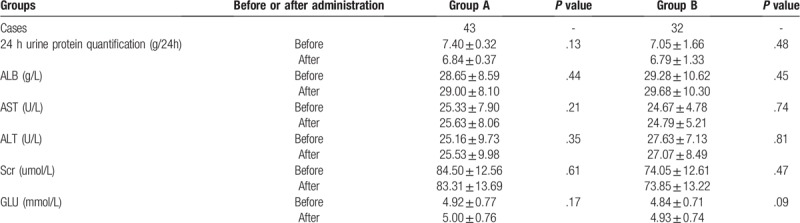
Comparisons of general condition in both groups before and after administration (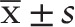).

### 3.2 Genotype distribution

Among the 75 patients, 6, 37 and 32 cases had the genotypes of CYP3A5∗1/∗1, CYP3A5∗1/∗3 and CYP3A5∗3/∗3, respectively, resulting in the genotype frequencies of 8.0%, 49.3% and 42.7%, respectively, which conformed to the Hardy-Weinberg equilibrium (*P* > .05). (Table 3).

**Table 3:**
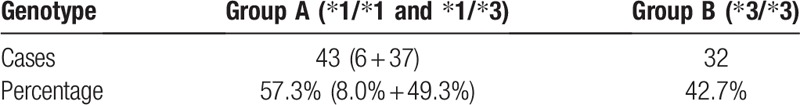
Genotype distribution and the proportion of 2 groups (%).

### 3.3 Effect of WZC on the FK506 blood concentration in both groups

Changes in the FK506 blood concentration before and after administration of WZC in groups A and B were compared. The results suggested that, the FK506 blood concentration in group A before taking WZC was (3.19 ± 0.73) ng/ml, while that was increased to (5.37 ± 0.81) ng/ml after taking WZC. In group B, the FK506 blood concentration before taking WZC was (6.43 ± 1.12) ng/ml, while that was increased to (8.19 ± 1.76) ng/ml after taking WZC. Such findings indicated that, the FK506 blood concentration was markedly elevated 1 week after taking WZC, and the difference before and after administration was statistically significant (*P* < .05) (Table 4).

**Table 4:**
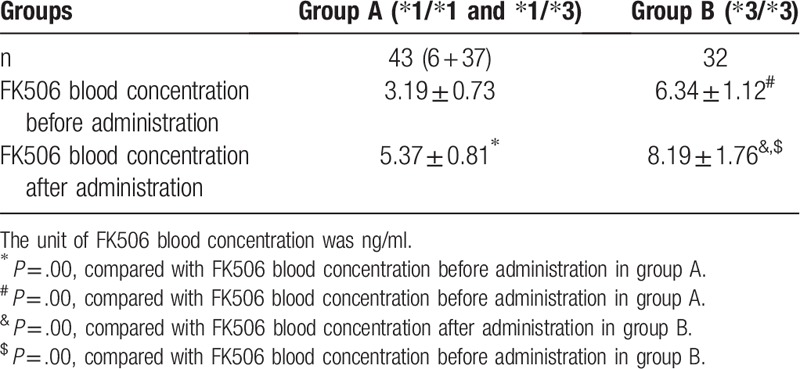
Comparison of FK506 blood concentration in both groups before and after administration (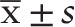).

### 3.4 Correlation of WZC on enhancing the early FK506 concentration in MN patients and the CYP3A5 gene polymorphism

**Figure 2:**
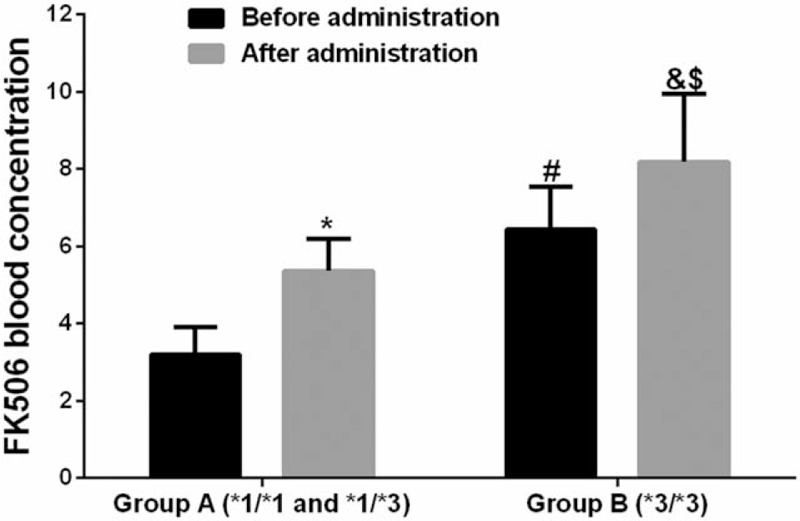
Histogram of the FK506 blood concentration changes in both groups before and after administration.

Difference in the FK506 blood concentration between groups A and B was compared, and the results suggested that, the FK506 blood concentration in group A before oral administration of WZC was lower than that in group B (*P* < .05). Meanwhile, the FK506 blood concentration in group A after oral administration of WZC was also lower than that in group B (*P* < .05), and the difference between two groups was statistically significant (*P* < .05) (Fig. 2).

## 4 Discussion

### 4.1 Effect of WZC on the FK506 blood concentration and general laboratory indexes

The blood concentration of patients enrolled in this study remained low after increasing the dose of tacrolimus. The difference in blood concentration between group A (CYP3A5 gene expression types, CYP3A5∗1/∗3 and CYP3A5∗1/∗1) and group B (CYP3A5 gene non-expression type (CYP3A5∗3/∗3) was compared, which suggested that, the FK506 blood concentration in group A before taking WZC was (3.19 ± 0.73) ng/ml, while that was increased to (5.37 ± 0.81) ng/ml after taking WZC. In group B, the FK506 blood concentration before taking WZC was (6.43 ± 1.12) ng/ml, while that was increased to (8.19 ± 1.76) ng/ml after taking WZC, and the difference before and after administration was statistically significant (*P* < .05) (Table 4). Of them, 10 patients had the FK506 blood concentration of ≥8.0 ng/mL 1 week after taking WZC, while 6 patients had that of ≥10 ng/ml. Thus, it could be observed that, WZC could effectively improve the FK506 blood concentration. Such conclusion was consistent with the results from existing studies.^[10–12]^ Tacrolimus can not only prevent the incidence of immune reaction, but can also treat the immune disease. Tacrolimus is a substrate of P-gp, which is located in the CYP3A metabolic enzyme in liver and small intestine.^[13]^ Drug interaction can induce and suppress the P-gp and CYP3A enzyme to affect the bioavailability and metabolism.^[14]^ It is reported that, both tacrolimus and deoxyschizandrin, the main active ingredient of WZC, are the substrates of CYP3A enzyme; moreover, WZC has contained multiple active ingredients that can suppress the activities of CYP450, P-gp and PXR. Compared with tacrolimus, deoxyschizandrin shows stronger affinity to CYP3A enzyme, which can competitively bind with CYP3A enzyme, reduce the binding of tacrolimus with CYP3A enzyme, reduce the transport of tacrolimus, and thus elevate the blood concentration of tacrolimus.^[15–18]^ Existing literature has proposed that, the metabolic process of midazolam is similar to that of tacrolimus in the body; before taking WZC, the CYP3A enzyme can metabolize midazolam into 1-hydroxyl midazolam to reduce its bioavailability; however, its bioavailability is markedly improved after taking WZC. Thus, WZC can suppress the CYP3A enzyme activity.^[19]^ In this study, differences in the 24 hours urine protein, ALB, Scr, ALT, AST and GLU in two groups [group A (CYP3A5 gene expression types, CYP3A5∗1/∗3 and CYP3A5∗1/∗1) and group B (CYP3A5 gene non-expression type (CYP3A5∗3/∗3)] were compared. The results revealed that, the 24 hours protein urine in groups A and B 1 week after administration was slightly reduced compared with that before administration, the ALB level was slightly increased, but the differences before and after administration were not statistically significant (*P* > .05). Meanwhile Scr, ALT and AST were not markedly changed before and after administration, and the differences were not statistically significant before and after administration (*P* > .05). As for GLU, it was slightly elevated after administration compared with that before administration, which might be related to the side effect of tacrolimus, but the difference before and after administration was not statistically significant (*P* > .05). Nonetheless, only the early clinical changes after taking WZC were observed in this study, which required further observation. Thus, it could be seen that, WAC combined with tacrolimus showed no obvious effect on the liver and kidney function.^[20]^

### 4.2 Effect of CYP3A5 gene polymorphism on the improvement of FK506 blood concentration by WZC

Cytochrome P450 (CYP) is involved in the metabolism and biotransformation of many drugs. Typically, CYP3A5 is one of the most important metabolic enzymes in the CYP enzyme system, which is the leading extrahepatic distribution form of the CYP3A subfamily. In the liver, CYP3A5 accounts for 20% of the CYP enzyme system in the liver, while the others are expressed in some organs, such as kidney, lung, prostate, intestinal wall, and some tumor tissues.^[21]^ CYP3A5 enzyme is located in chromosome 7, with the gene full length of 31.8 kb; it contains 13 exons and encodes 502 amino acids.

The 6986-position A on intron 3 (rs776746) of GYP is mutated into G, and such gene mutation will result in the early appearance of termination codon on the 109 site during RNA transcription. As a result, the translated protein fragment has no function, such enzyme is not expressed in patients carrying such gene, and such mutation will render reduced or disappeared activity of the CYP3A5 enzyme. The wild-type CYP3A5 is defined as CYP3A∗1. As suggested in research, normal protein expression can only be carried out when an individual has carried at least one CYP3A5∗1 allele.^[22]^ In this experiment, the proportion of CYP3A5∗1 allele was 57.3%, which was slightly higher than that reported by Hou Mingming et al.^[23]^ Our results suggested that, the initial blood concentration of tacrolimus in patients with CYP3A5∗3/∗3 type was notably higher than that in patients with CYP3A5∗1/∗1 type and CYP3A5∗1/∗3 type after taking equivalent doses of tacrolimus, which was basically consistent with the results by Onizuka.^[24]^ It was suggested in this study that, patients with CYP3A5∗3/∗3 type would not express CYP3A5 enzyme, which would reduce its activity, decrease the metabolism of tacrolimus, and thus elevate its blood concentration.

A total of 75 patients were enrolled in this study, and divided according to the metabolic enzyme expression of CYP3A5 gene into group A (CYP3A5∗1/∗1 type and CYP3A5∗1/∗3 type), and group B (CYP3A5∗3/∗3 type). Among them, 43 cases were in group A, accounting for 57.3%, and 32 were in group B, taking 42.7%. Findings in this study were basically consistent with the research data of CYP3A5 expression among the Asian population. In this study, the blood concentration of tacrolimus in group A before administration was markedly lower than that in group B; after administration, the blood concentration of tacrolimus in group A was also evidently lower than that in group B, and the difference was statistically significant (*P* < .05). It is reported in literature that, CYP3A5 gene polymorphism will dramatically affect the blood concentration of tacrolimus in patients receiving renal transplantation. Particularly, there is great difference between patients with CYP3A5 enzyme expression types (∗1/∗1 and ∗1/∗3 types) and CYP3A5 enzyme non-expression type (∗3/∗3), and the latter is 1.8–3.3 folds higher than that in the former. Besides, the blood concentration of ∗1/∗1 type is slightly lower than that of ∗1/∗3 type, but the difference is not statistically significant.^[25,26]^ At present, many studies at home and abroad have reported that, CYP3A5 gene polymorphism will affect the blood concentration of tacrolimus.^[27–30]^ Our results conformed to those reported at home and abroad before.

As found in this study, the blood concentration of tacrolimus in patients with CYP3A5 gene expression types (∗1/∗1 type and ∗1/∗3 type) was lower than that in patients with CYP3A5 gene non-expression type (∗3/∗3) after taking the same dose of tacrolimus (mg/kg) orally. In other words, patients with CYP3A5∗1/∗1 and ∗1/∗3 types have to take tacrolimus at a higher dose than patients with CYP3A5∗3∗3 type to achieve the same blood concentration of tacrolimus.

A variety of factors and drugs will have certain influence on the metabolism of tacrolimus, apart from CYP3A5 gene polymorphism and WZC, such as CYP3A4 and MDR1 gene polymorphisms, as well as the commonly used glucocorticoid and calcium channel blocker in clinic. However, the mechanism has not been illustrated yet. In addition, the observation time in this experiment is relatively short, and only early clinical observation is carried out. Therefore, the influence of the above several factors on tacrolimus in clinic will be further examined in future studies. Besides, this is a single-center study, with a small sample size and short follow-up length. Thus, in future studies, large sample size, long-term and multi-center experimental research will be carried out.

## 5 Conclusion

The gene polymorphism of CYP3A5∗3 can affect the metabolism of tacrolimus, and a typing shows lifelong benefits. When WZC is used in combined with tacrolimus, the FK506 blood concentration can be markedly improved. The FK506 concentration in patients with CYP3A5∗1/∗1 and CYP3A5∗1/∗3 types is lower than that in patients with CYP3A5∗3/∗3 type, when equivalent doses of tacrolimus are given.

## Author contributions

**Data curation:** Xiaobei Lu.

**Formal analysis:** Xiaobei Lu.

**Funding acquisition:** Zhu Zhang.

**Methodology:** Zhu Zhang, Leipeng Dong.

**Project administration:** Zhu Zhang.

**Resources:** Xiaoguang Fan.

**Software:** Leipeng Dong, Jiwei Ma.

**Writing – original draft:** Zhu Zhang, Jiwei Ma.

**Writing – review & editing:** Zhu Zhang, Xiaoguang Fan.

Zhu Zhang orcid: 0000-0002-3359-3995.
